# Prevalence and risk factors of malaria among first antenatal care attendees in rural Burkina Faso

**DOI:** 10.1186/s41182-022-00442-3

**Published:** 2022-07-25

**Authors:** Moussa Lingani, Serge H. Zango, Innocent Valéa, Maïmouna Sanou, Serge Ouoba, Sékou Samadoulougou, Annie Robert, Halidou Tinto, Michèle Dramaix, Philippe Donnen

**Affiliations:** 1 École de Santé Publique, Université Libre de Bruxelles, Bruxelles, Belgique; 2 Institut de Recherche en Sciences de la Santé/Direction Régionale du Centre Ouest (IRSS/DRCO), Nanoro, Burkina Faso; 3 Epidemiology and Biostatistics Research Division, Institut de Recherche Expérimentale et Clinique, Université Catholique de Louvain, Bruxelles, Belgique; 4 Evaluation Platform on Obesity Prevention, Quebec Heart and Lung Institute Research Center, Quebec, Canada

**Keywords:** Malaria, Pregnancy, First antenatal care visit, Burkina Faso

## Abstract

**Background:**

The WHO recommends continuous surveillance of malaria in endemic countries to identify areas and populations most in need for targeted interventions. The aim of this study was to assess the prevalence of malaria and its associated factors among first antenatal care (ANC) attendees in rural Burkina Faso.

**Methods:**

A cross-sectional survey was conducted between August 2019 and September 2020 at the Yako health district and included 1067 first ANC attendees. Sociodemographic, gyneco-obstetric, and medical characteristics were collected. Malaria was diagnosed by standard microscopy and hemoglobin level was measured by spectrophotometry. A multivariate logistic regression analysis was used to identify factors associated with malaria infection.

**Results:**

Overall malaria infection prevalence was 16.1% (167/1039). Among malaria-positive women, the geometric mean parasite density was 1204 [95% confidence interval (CI) 934–1552] parasites/µL and the proportion of very low (1–199 parasites/µL), low (200–999 parasites/µL), medium (1000–9999 parasites/µL) and high (≥ 10,000 parasites/µL) parasite densities were 15.0%, 35.3%, 38.3% and 11.4%, respectively. Age < 20 years (adjusted odds ratio (aOR): 2.2; 95% CI 1.4–3.5), anemia (hemoglobin < 11 g/deciliter) (aOR: 3.4; 95% CI 2.2–5.5), the non-use of bed net (aOR: 1.8; 95% CI 1.1–2.8), and the absence of intermittent preventive treatment with sulfadoxine–pyrimethamine (aOR: 5.8; 95% CI 2.1–24.5) were positively associated with malaria infection.

**Conclusions:**

The study showed that one out of six pregnant women had a microscopy-detected *P. falciparum* malaria infection at their first ANC visit. Strengthening malaria prevention strategies during the first ANC visit is needed to prevent unfavorable birth outcomes.

## Introduction

Despite efforts to control and eliminate malaria, it remains a major public health concern for sub-Saharan African (SSA) populations, particularly pregnant women and children under 5 years of age [1]. Indeed, malaria in pregnancy (MiP), caused by *Plasmodium* parasites, can lead to severe pregnancy outcomes such as maternal and fetal anemia, stillbirth, intrauterine growth retardation and preterm births, with the latter two being major contributors to low birth weight (LBW), a strong precursor for neonatal mortality [2–4]. In 2020, there were an estimated 11.6 million (34%) pregnancies exposed to malaria infection in the World Health Organization (WHO) African Region, which resulted in 900,000 LBW, 25,000 maternal deaths, and 100,000 neonatal deaths [1].

To control malaria and mitigate its adverse effects on pregnancy outcomes, preventive strategies have been recommended by the WHO, including the use of insecticide-treated nets (ITNs) and the intermittent preventive treatment in pregnancy with sulfadoxine–pyrimethamine (IPTp-SP) [1]. In Burkina Faso, IPTp-SP coverage reached 77% and ITN coverage was 93% in 2020 [5]. Despite these efforts, malaria remains frequent and unevenly distributed in sub-Saharan Africa, and its presence can be considered an indirect indicator of inadequate prevention strategies [1].

As the malaria burden greatly varies in different geographical settings and populations, the WHO has recommended continuous surveillance of malaria cases to identify areas and populations most in need, so that appropriate resources and targeted interventions can be implemented and their impact monitored and evaluated [1, 6].

In Burkina Faso, the epidemiology of malaria has been comprehensively described for different age groups and conditions [7–11], and various interventions have been implemented to improve health care [12–14]. However, data on the characteristics of malaria in pregnancy are sparse, particularly in rural areas, and it is an obstacle for adequate control strategies planning and implementation. The aim of this study was to characterize the burden of malaria infection and to identify its associated factors among pregnant women during their first ANC visit in the rural area of Burkina Faso. This could help update the antenatal care package provided to pregnant women at their first ANC visit, thus reducing the adverse effects of malaria on pregnancy outcomes.

## Methods

### Study area and design

This was an ancillary study of a clinical trial comparing the efficacy of IPTp-SP versus IPTp-SP plus azithromycin to prevent low birth weight (www.pactr.org, registration number PACTR201808177464681) in the health district of Yako, rural Burkina Faso. This study consisted of a cross-sectional survey of pregnant women attending their first antennal care visit during their ongoing pregnancies. Participants were enrolled at three peripheral health centers of the Yako health district. In Yako, malaria transmission is holoendemic, with peak transmission occurring during the rainy season (July–November) [15]. The health district catchment area covers 424,577 inhabitants, and 23,000 pregnancies were recorded in 2017 [15]. Also, malaria represented the main reason for consultation, hospitalization and death, with the highest burden carried by children under 5 years of age and pregnant women [15].

### Inclusion/exclusion criteria

Pregnant women aged 16 to 45 years, with a gestational age ≤ 24 weeks, mainly residing in the health district catchment area, and willing to participate in the study were requested to provide their written informed consents before they were enrolled. Exclusion criteria included any inability to adhere to the study procedures.

### Data collection procedures

At enrollment, pregnant women were clinically examined to collect sociodemographic, obstetric, medical characteristics, and malaria prevention measures. Data were obtained by interview of the pregnant women and recorded onto semi-structured questionnaires by trained nurses. Age, gyneco-obstetrical history, IPTp-SP uptake before the first ANC visit, educational level, occupation, and the use of bed nets the night before antenatal clinic visit were collected. In addition to physical and obstetrical examinations, blood pressure, axillary temperature, and body weight were measured, completed by malaria diagnosis in peripheral blood samples. Due to the absence of ultrasounds, we estimated gestational age using the knowledge of the last menstrual period (LMP), or through the symphysial fundal height (SFH) measurement whenever the LMP was unknown. Body weights were measured with calibrated Seca 813—scales with a precision of 100 g (Seca gmbh & co. kg, Germany). The dependent variable was a microscopy-detected malaria infection, defined as any density of asexual malaria parasite during the microcopy examination of thick and thin blood smears (Table 1).

**Table 1:** List and definition of variables

	Variables	Definitions	Categories
1	Health facility	Place of recruitment	District 4, district 5 and district 6
2	Age	Age of women (in years)	< 20, ≥ 20
3	Educational level	Educational level (years of schooling)	None (0 years), primary (1–6 years), secondary—plus (> 6 years)
4	Occupation	Occupation of the woman	Unemployed, employed/self-employed
5	Marital	Marital status	Single, married or living together
6	Residency	Residence of the participant	Semi-urban, rural
7	Gestational age	Gestational age at first ANC	< 20 weeks, ≥ 20 weeks
8	Gestity	Number of pregnancies	1, 2, 3 or more
9	Miscarriage/stillbirth	History of stillbirth or miscarriage	Yes/no
10	Body weight	Woman's body weight (in kilograms)	In kilograms (± 100 g)
11	ITN use	Use of ITN the night before the visit	Yes/no
12	IPTp-SP	Number of IPTp-SP doses received	1 dose, 0 dose
13	Anemia	Hemoglobin level < 11 g/dL	No/yes
14	Malaria infection	Positive blood smear at microscopy	No/yes

ANC: antenatal care, ITN: Insecticide-treated bed net, IPTp-SP: intermittent preventive treatment of malaria in pregnancy using sulfadoxine–pyrimethamine

### Laboratory procedures

Trained nurses collected peripheral blood samples by finger pricks and prepared thick and thin blood smears, which were subsequently shipped to the Nanoro clinical laboratory for standard microscopy. Thick blood smears were used to detect the presence of malaria parasites, while thin blood smears were used to discriminate the *Plasmodium* species that caused the infection. The slides were stained with 5% Giemsa for 30 min and independently double examined by two certified microscopists assuming a white blood cell count of 8000/µL*.* In case of discrepancy (discrepant species or count difference of at least 50%), a third independent reading was done. The final result was the average of the two closest results.

### Sample size calculation

In Burkina Faso, the prevalence of malaria among pregnant women at predelivery in the rural area was estimated at around *p* = 17.5% [5]. With the hypothesis that this prevalence would be similar or higher during the first ANC visit, the required sample size was calculated by using the Cochran formula *n* = *Z*^2^ *p*(1 − *p*)/*i*^2^, where *p* is the expected proportion, *i* = 2.5%, the margin of error, and *Z* corresponds to the 95% confidence interval (1.96). With a 10% margin of missing data, approximately 1000 participants were required.

## Data processing and analysis

Data were collected on a semi-standardized questionnaire, double entered onto the OpenClinica software, and exported onto RStudio (Version 1.2.5042) for cleaning and analysis. Participants were grouped as adolescents (below 20 years of age) or adults (20 years or more), and frequency tables were used to summarize categorical variables. Mean or median with respective standard deviations or quartiles were used to summarize numerical variables. To investigate factors associated with malaria, we conducted a univariate logistic analysis to calculate odds ratios (OR) and their 95% confidence intervals (95% CI). All variables with a *p*-value below 0.10 at univariate analysis were included in the starting model of a multivariate analysis. Then, the variables were eliminated step-by-step using the backward selection procedure. At this level, only variables for which the *p*-value was less than 0.05 were kept in the final model. The number of pregnancies was excluded from the multivariate analysis due to its strong correlation with the women's age. The significance level was set at 5% (two-sided *p*-value).

## Results

### Study participants' sociodemographic, obstetric, and malaria prevention characteristics at enrollment

A total of 1067 first antenatal care attendees were screened from three peripheral health centers: 616 (57.7%) from the district Nº4, 254 (23.8%) from the district Nº5, and 197 (18.5%) from the district Nº6. The mean age was 25.0 (± 5.7) years, and nearly 15.0% of them were of young age (age below 20 years). Most of them were not involved in any income-generating activities (81.8%). The primigravid and secundigravid women represented 30.3% and 22.4% of the study population, respectively, with a mean gestational age of 21.6 (± 2.2) weeks. About 42.5% did not have any formal education, and up to 42.2% completed high school or higher educational level. Nearly 80.0% declared sleeping under an ITN the night before they visited the health facility, and almost 9.0% of them reported taking a SP dose before their first ANC visit. Study participants' sociodemographic, gyneco-obstetric, and medical characteristics are summarized in Table 2.

**Table 2:** Characteristics of first ANC attendees in the Yako health district, Burkina Faso (*n* = 1067)

Characteristics	Mean (± SD) or *n* (%)
Age (in years), mean (± SD)	25.0 (± 5.7)
< 20, *n* (%)	155 (14.6)
≥ 20, *n* (%)	910 (85.4)
Level of education	
None, *n* (%)	452 (42.4)
Primary, *n* (%)	165 (15.5)
Secondary or more, *n* (%)	450 (42.2)
Occupation	
Unemployed, *n* (%)	871 (81.8)
Employed/self-employed, *n* (%)	194 (18.2)
Marital status	
Single, *n* (%)	54 (5.1)
Married or living together, *n* (%)	1013 (94.9)
Sleeping under an ITN the night before the visit	
No, *n* (%)	215 (20.1)
Yes, *n* (%)	852 (79.9)
Uptake of IPTp-SP dose	
No, *n *(%)	974 (91.3)
Yes, *n* (%)	93 (8.7)
Gravidity	
Primigravidae (1st pregnancy), *n* (%)	323 (30.3)
Secundigravidae (2nd pregnancy), *n* (%)	240 (22.5)
Multigravida (3rd pregnancy or more), *n* (%)	504 (47.2)
Gestational age at first ANC visit (weeks), mean (± SD)	21.6 (± 2.2)
< 20, *n* (%)	122 (12.3)
> 20, *n* (%)	870 (87.7)
History of stillbirth or miscarriage	
Yes, *n* (%)	165 (15.5)
No, *n* (%)	902 (84.5)
Body weight (kg), mean (± SD)	61.7 (± 10.5)
< 50, *n* (%)	71 (7.0)
> 50, *n* (%)	948 (93.0)
Hemoglobin level (g/dL), mean (± SD)	10.5 (± 1.4)
≥ 11, *n* (%)	320 (40.0)
< 11, *n* (%)	481 (60.0)
Microscopy-detected malaria infection	
No, *n* (%)	872 (83.9)
Yes, *n* (%)	167 (16.1)

ITN: insecticide-treated bed net, g/dL: gram per deciliter, ANC: antenatal care; IPTp-SP: intermittent preventive treatment in pregnancy with sulfadoxine–pyrimethamine; g/dL: gram per deciliter, SD: standard deviation; %: percentage

Overall, 16.1% (167/1039) (95% CI 13.4–18.5%) of participants had a microscopy-detected malaria infection. The prevalence of malaria was significantly higher among women aged below 20 years (28.8%, [95% CI 21.9%–36.7%]) compared to those ≥ 20 years (13.8%, [95% CI 11.6–16.3%]) (*p*-value = 0.01). The prevalence of malaria infection was nearly twice as high among women who did not use an ITN prior their first ANC visit (22.5% [95% CI 17.1–28.9%]) compared to those who used an ITN (14.5% [95% CI 12.2–17.1%]), with however a non-significant difference (*p* = 0.19). Women without any dose of SP before the visit had a higher proportion of malaria infections (17.2% [95% CI 14.9–19.8%]) compared to those who had a dose of SP (4.3% [95% CI 1.4–11.4%], *p* = 0.01). *Plasmodium falciparum* was the only species detected during the thin blood smear examination. The geometric mean of parasite density was 1204 [95% CI 934–1552] parasites/µL. The proportion of very low (1–199 parasites/µL), low (200–999 parasites/µL), medium (1000–9999 parasites/µL) and high (≥ 10,000 parasites/µL) parasite densities were 15.0%, 35.3%, 38.3% and 11.4%, respectively. The mean hemoglobin level was 10.5 (± 1.4) g/dL, and nearly 60.0% of women were anemic (hemoglobin level < 11.0 g/dL) at their first ANC visit.

### Factors associated with microscopy-detected malaria infection

In the unadjusted univariate analysis, the young maternal age, the presence of maternal anemia, the absence of ITN use, and the non-usage of sulfadoxine–pyrimethamine were significantly associated with the presence of malaria infection (*p* < 0.05) (Table 3). After adjustment with other covariates using the multivariate logistic regression, the younger age (< 20 years) (adjusted OR (aOR) = 2.2, [95% CI 1.4–3.5]), the presence of anemia (aOR = 3.4, [95% CI 2.2–5.5]), the lack of ITN use the night before the first ANC visit (aOR = 1.8, [95% CI 1.2–2.8]), and the non-uptake of sulfadoxine–pyrimethamine before the first ANC visit (aOR = 5.8, [95% CI 2.1–24.5]) were independently associated with the presence of a microscopy-detected malaria (Table 3).

**Table 3:** Bivariate and multivariate analyses of factors associated with malaria infection among first ANC attendees (*n* = 1039) in the Yako health district, rural Burkina Faso

Characteristics	*N*	Malaria infection *n* (%)	OR [95% CI]	*p*-value	aOR^1^ [95% CI]	*p*-value
Overall	1037	166 (16.0)	–	–	–	–
Age group (in years)						
< 20	153	44 (28.8)	2.5[1.7–3.7]	0.001	2.2 [1.4–3.5]	0.001
≥ 20	884	122 (13.8)	Ref		Ref	
Formal education						
No formal education	439	62 (14.1)	0.8 [0.5–1.1]	0.143	–	–
Formal education	600	105 (17.5)	Ref			
Occupation						
Unemployed	848	144 (17.0)	1.5 [0.9–2.4]	0.105	–	–
Employed/self-employed	189	23 (12.2)	Ref			
Marital status						
Single	53	13 (24.5)	1.8 [0.9–3.3]	0.089	–	–
Married or living together	986	154 (15.6)	Ref			
Residency						
Rural	95	18 (18.9)	1.2 [0.7–2.1]	0.42	–	–
Semi-rural	944	149 (15.8)	Ref	–		
Hemoglobin (in grams/deciliter)						
< 11	476	113 (23.7)	3.5 [2.2–5.6]	< 0.001	3.4 [2.2–5.5]Ref	< 0.001
≥ 11	316	26 (8.2)	Ref			
ITN use						
No	209	47 (22.5)	1.7 [1.2–2.5]	0.005	1.8 [1.2–2.8]	0.009
Yes	830	120 (14.5)	Ref		Ref	
IPTp-SP (doses)						
0	947	163 (17.2)	4.6 [1.8–15.1]	0.003	5.8 [2.1–24.5]	0.003
1	92	4 (4.3)	Ref		Ref	

CI: confidence interval; OR: odds ratio; aOR: adjusted odds ratio; ITN: insecticide-treated bed net; g/dL: gram per deciliter; IPTp-SP: intermittent preventive treatment in pregnancy with sulfadoxine–pyrimethamine

^1^Variable age group, occupation, marital status, anemia, ITN use the night before antenatal clinic visit, and uptake of a dose of sulfadoxine–pyrimethamine were included in the multivariate logistic regression model

## Discussion

Nearly one out of six pregnant women attending their first antenatal clinic visit for their ongoing pregnancy had a microscopy-detected malaria infection in our study setting. This suggests that prevention strategies should be strengthened as malaria infection in early pregnancy is associated with a higher risk of unfavorable birth outcomes [16, 17]. The prevalence of microscopy-detected malaria in our study was similar to that reported at predelivery in the same area, with a prevalence of 17.5% (95% CI 14.6–20.8%). A similar trend was reported in the central region of the country (a similar malaria transmission setting), with a prevalence of microscopy-detected *P. falciparum* infection of 15.7% (95% CI 12.3–20.2%) during the second and third trimesters of pregnancy [7]. This shows that malaria transmission intensity in the country is consistent throughout the pregnancy [5, 10]. Thus, initiating and maintaining prevention methods during the whole course of the pregnancy would be necessary to control the adverse effects of malaria infection on birth outcomes. Much higher figures were reported in some African countries, with nearly 20.4% in the middle belt of Ghana [18], 25.4% in Benin [19], 59.7% in Ghana (Navrongo), 42.2% in Mali, and 16.8% in The Gambia [20] between pregnancy conception and first ANC contact. These figures decreased during the low transmission season, with 41.3%, 34.4%, 11.5%, and 7.8%, in Navrongo (Ghana), Mali, Benin, and The Gambia, respectively [20]. This variation of malaria prevalence across different settings is likely related to differences in malaria transmission intensity, malaria prevention measures used and coverage, and environmental patterns [21–24]. This variation could also be related to the variation of protective immunity levels according to the geographic area and the number of pregnancies of the woman [25–27]. Thus, it is necessary to implement and monitor targeted interventions adapted to each setting and type of population to ensure adequate prevention of malaria and its negative impact on birth outcomes.

The geometric mean parasite density was much higher than that reported in Ghana (442 parasites/µL) and Cameroon (529 parasites/µL) [28]. However, similar findings were reported in other settings, such as Zambia, with 1082 parasites/µL [29]. These variations can be explained by the difference in transmission intensities and the overall coverage of protective methods such as the use of ITN, indoor residual sprays, and other preventive measures. This can also be related to the approach to calculate parasite density, either using the absolute white blood cell (WBC) count of the patient or assuming a WBC count of 8000 cells/µL, and the latter may overestimate the parasite densities [30–32]. As peripheral blood parasite density also correlates well with placental malaria, a major determinant of poor feto-maternal nutrients exchange, particular attention is needed in our setting, where pregnant women's parasite densities tend to be higher than in other sub-Saharan African settings [33, 34].

*P. falciparum* was the only species reported in this study. Such a result was not surprising and corroborates previous findings that reported *P falciparum* as the main species involved in malaria in the country [8]. This could explain why more than 95% of the malaria burden is reported in sub-Saharan Africa, as *P. falciparum* is the deadliest malaria parasite and the most prevalent on the African continent [35]. Thus, a reinforcement of *P. falciparum* infection prevention measures is needed to control malaria’s impact on pregnancy outcomes.

The proportion of women that used an ITN the night prior to their visit to the antenatal clinic was surprisingly high (80%), given that ITN distribution to pregnant women usually occurs at the first ANC visit. This may be because almost 70% of the pregnant women were secundigravid or more, suggesting they may have already been exposed to ANC service and received ITN. The prevalence of malaria infection was significantly higher among women who did not use an ITN the night before they visited the antenatal clinic. This finding is in line with previous reports demonstrating the effectiveness of ITN in preventing malaria in pregnancy [36]. Pre-conception ITN supply to women could be key to increasing the coverage rate at the first ANC visit in our setting.

Although SP use is not indicated during the first trimester of pregnancy, nearly one out of ten pregnant women had their first dose prior to their first ANC visit, and the risk of malaria infection was lower among women who had SP before the first ANC. Given that the mean gestational age at first ANC was nearly 22 weeks in our study, strategy is thus needed to cover pregnant women as early as 12 to 16 weeks of gestation to benefit from the full potential of the IPTp-SP strategy [37]. In addition, interventions that could protect pregnant women early in their pregnancy, when most of the available methods are contraindicated, are needed.

In this study, adolescent pregnant women were found to have a higher prevalence of malaria infection with up to a twofold increase. This is in line with studies conducted in different sub-Saharan African countries which reported that young pregnant women are at the greatest risk of malaria infection and have the highest parasite densities [18, 38, 39]. This may be attributed to the fact that adolescent pregnant women did not have adequate exposure to health services and did not gain a good awareness about malaria and its prevention approaches. Also, due to previous frequent malaria exposures, older pregnant women might have developed protective immunity to malaria infection [25–27]. Pre-conceptional exposure of adolescent women to health services would increase their awareness at the time of their pregnancy and thus facilitate early adoption of preventive measures.

In this cross-sectional survey, we described the prevalence of malaria infection during the first ANC contact in a rural health district of Burkina Faso. However, some limitations are worth noting. Indeed, pregnant women were not followed up to evaluate the effect of malaria infection during the first ANC visit on birth outcomes. In addition, gestational age was assessed using the pregnant women's knowledge of their last menstrual period or the fundal height measurement in most cases due to the absence of ultrasounds, and this is frequently prone to many errors. Furthermore, the standard microscopy diagnosis approach used for this study may have underestimated the actual prevalence, considering that submicroscopic malaria infections are common in endemic areas. However, the study has highlighted the burden of malaria infection at the first ANC contact among pregnant women and suggested strategies to mitigate malaria's impact on pregnancy outcomes.

## Conclusion

The study showed that one out of six pregnant women had malaria infection at their first antenatal care visit. This rate was even higher among pregnant adolescents, with one in three cases of malaria infection at their first ANC. Adequately preventing infection during that period of the pregnancy could substantially benefit pregnant women and their unborn fetuses.

## Data Availability

The datasets used and/or analyzed during the current study are available from the corresponding author on reasonable request.

## Abbreviations

ANC: Antenatal care
OR: Odds ratios
aOR: Adjusted odds ratios
Hb: Hemoglobin
HBP: High blood pressure
IPTp-SP: Intermittent treatment of malaria in pregnancy using sulfadoxine–pyrimethamine
ITNs: Insecticide-treated nets
LBW: Low birthweight
WHO: World Health Organization
